# Cyclic Alternating Patterns of Encephalopathy (CAPE) in Acute Brain Injury Through a Quantitative Electroencephalogram (QEEG) Perspective: A Case Series

**DOI:** 10.7759/cureus.77436

**Published:** 2025-01-14

**Authors:** Leopoldo Cendejas-Zaragoza, Christopher R Newey, Marvin A Rossi, Harrison Wood, Madihah Hepburn

**Affiliations:** 1 Neurology, Comprehensive Epilepsy Center, Summa Health, Akron, USA; 2 Neurocritical Care, Sanford USD Medical Center, Sioux Falls, USA; 3 Neurology, Neurocritical Care, Summa Health, Akron, USA

**Keywords:** acute brain injury, cyclic alternating pattern of encephalopathy, eeg, encephalopathy, quantitative eeg

## Abstract

Continuous EEG (cEEG) is a non-invasive bedside tool used to detect causative or contributory conditions of the encephalopathic state. By continuously recording electrical brain activity, it provides insights into background patterns, seizures, and dynamic cerebral activity, thereby aiding in the management of critically ill patients with acute brain injury.

The term 'cyclic alternating pattern of encephalopathy' (CAPE) was recently introduced to describe alternating changes in brain electrical activity observed on EEG in critically ill patients. CAPE is characterized by electrocerebral background pattern shifts lasting at least ten seconds and repeating regularly for a minimum of six cycles.

Quantitative EEG (QEEG) facilitates the interpretation of extensive cEEG datasets by applying mathematical algorithms to transform raw EEG data into time-compressed, frequency- or amplitude-based visualizations. Through Fourier analysis, QEEG decomposes the EEG signals, plotting the amplitude of different frequency bands over time, enabling easier identification of state changes such as CAPE across extended periods.

This case series highlights four critically ill patients exhibiting CAPE on cEEG, with corresponding findings illustrated via QEEG. These cases demonstrate that QEEG effectively identifies CAPE by detecting changes in spectral power density and rhythmicity across distinct states. Adjusting the temporal resolution on QEEG enhances the visibility of CAPE patterns, facilitating their recognition.

## Introduction

Acute encephalopathy, an acquired state of global cognitive dysfunction, is commonly observed in critically ill patients admitted to ICUs. Clinically, it manifests as a spectrum of arousal impairments, ranging in severity from hypoactive and hyperactive delirium to coma. This condition reflects acute brain dysfunction caused by an underlying pathophysiological process [1]. The etiology of acute encephalopathy is often multifactorial, with contributors including medications (e.g., sedative infusions, analgesics), illicit drug use, systemic illnesses leading to cytokine release and cerebral dysfunction (e.g., sepsis, hepatic encephalopathy), and metabolic derangements (e.g., renal failure, electrolyte disturbances). Seizures, particularly non-convulsive seizures (NCS), are a common cause of encephalopathy, occurring in up to 17.9% of critically ill patients [2-4]. Acute brain injuries, such as stroke or intracranial hemorrhage, can also directly contribute or worsen encephalopathy [5].

Identifying the contributors to encephalopathy is critical, as complications can prolong ICU stays and increase the risk of secondary brain injury [6,7]. Continuous EEG (cEEG), a non-invasive bedside tool, plays a crucial role in detecting causative or contributory conditions of the encephalopathic state. cEEG provides information on background activity, sleep architecture, epileptiform discharges, state changes, rhythmic patterns, and reactivity to external stimuli [8].

In 2021, the American Clinical Neurophysiology Society (ACNS) Standardized Critical Care EEG terminology introduced the concept of cyclic alternating pattern of encephalopathy (CAPE) as a new term defined by changes in electrocerebral background patterns each lasting at least ten seconds and spontaneously alternating regularly for at least six cycles [9]. CAPE differs from “reactivity” which denotes changes in cerebral activity to external stimulation [10,11]. While EEG reactivity has been linked to prognostic value, the clinical significance of CAPE in the critically ill patient remains unclear [12].

The use of cEEG in the diagnosis and management of encephalopathy in critically ill patients involves prolonged monitoring over hours to days to capture the progression or resolution of encephalopathy. This results in extensive raw EEG recordings that require significant time and expertise to interpret. To aid in the analysis of these large datasets, quantitative EEG (QEEG) applies mathematical algorithms to convert raw EEG signals into time-compressed visual representations, such as spectrograms and rhythmicity plots, which summarize brain activity patterns over extended timeframes.

QEEG encompasses a variety of analytical techniques that decompose EEG signals to highlight changes in brain activity. One commonly used approach is Fourier analysis, which transforms EEG signals into their constituent frequency components and plots their amplitude over time. This enables the identification of dynamic state transitions, including CAPE. By presenting EEG data in summary trends, QEEG facilitates the detection of subtle yet clinically significant changes that may otherwise be difficult to discern in raw EEG recordings.

While CAPE has been well-characterized in raw EEG data, its representation across QEEG modalities has not been thoroughly investigated. The objective of this case series is to illustrate CAPE patterns using QEEG and to demonstrate how QEEG can enhance the recognition of these alternating states in encephalopathic patients.

## Case presentation

This case series presents four critically ill patients with diverse acute brain insults, all exhibiting a CAPE on cEEG monitoring. Table 1 summarizes the admission laboratory findings for each case, offering a comparative perspective on systemic factors contributing to their encephalopathy.

**Table 1:** Admission labs ALP: Alkaline phosphatase; ALT: Alanine aminotransferase; AST: Aspartate aminotransferase; BUN: Blood urea nitrogen; CK: Creatine kinase; Creatinine: Serum creatinine; Glucose: Blood glucose level; HGB: Hemoglobin; INR: International normalized ratio; Lactic acid: Blood lactic acid level; NA: Not available during admission; pCO₂: Partial pressure of carbon dioxide in arterial blood; pH: Arterial blood pH; Platelets: Platelet count; T Bilirubin: Total bilirubin; Troponin: Cardiac troponin level; WBC: White blood cell count

Parameter	Case #1	Case #2	Case #3	Case #4	Reference Ranges (units)
Subject description	29 y.o. male	71 y.o. female	32 y.o. male	72 y.o. female	
WBC	25.2	9.7	21.7	10.4	3.6-10.7 (10^3^/uL)
HGB	10.3	11.1	15.0	12.9	Men: 13.0-18.0 (g/dL); Women: 11.7-16.0 (g/dL)
Platelets	148	16	347	188	140-400 (10^3^/uL)
INR	2.1	NA	NA	4.4	0.9-1.1
Sodium	139	136	138	130	135-145 (mmol/L)
BUN	55	26	20	67	9-20 (mg/dL)
Creatinine	2.33	0.69	2.56	4.38	0.6-1.25 (mg/dL)
Glucose	155	136	133	110	70-100 (mg/dL)
Arterial pCO_2_	27.4	30.4	33.4	33.1	35-45 (mmHg)
Arterial pH	7.461	7.499	7.459	7.438	7.350-7.450
Lactic acid	5.7	3.8	6.8	2.2	0.7-2.0 (mmol/L)
Troponin	8.37	<0.012	0.149	0.022	<0.034 (ng/mL)
AST	255	NA	432	125	15-46 (U/L)
ALT	134	NA	364	142	0-49 (U/L)
ALP	228	NA	110	86	38-126 (U/L)
T bilirubin	1.2	NA	0.3	2.1	0.2-1.3 (mg/dL)
CK	1,078	NA	604	117	30-170 (U/L)

Case 1

A 29-year-old male with opioid use disorder (OUD) and intravenous drug use (IVDU) was found unresponsive at home. Emergency medical services (EMS) administered naloxone, but his mental status did not improve. Right gaze deviation and left hemiplegia were noted. He was brought to the hospital as a stroke activation with a National Institutes of Health Stroke Scale (NIHSS) score of 33, consistent with right middle cerebral artery (RMCA) syndrome secondary to a large vessel occlusion (LVO). The severity of NIHSS and his comatose state was also concerning for NCS in addition to acute stroke.

**Figure 1:**
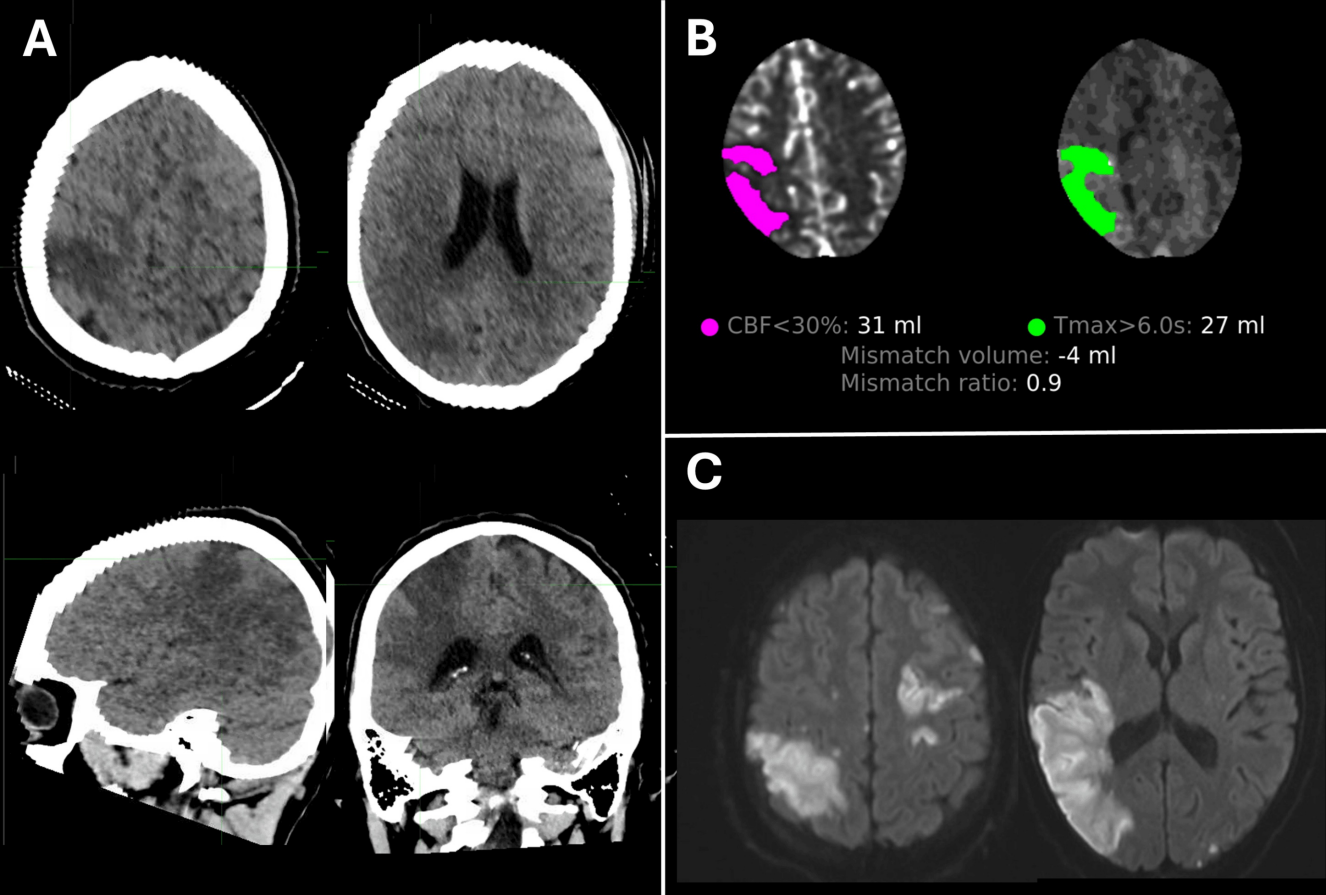
(Case 1) Neuroimaging (A) Non-contrast CTH demonstrates wedge-shaped hypoattenuation in the right MCA territory, consistent with acute infarction. (B) CTP quantitative analysis reveals a core infarct (CBF < 30%, volume = 31 mL) with no significant ischemic penumbra (tissue at risk volume (T_max_ > 6 seconds) = 27 mL). (C) DWI, performed one day later, shows diffusion restriction abnormalities indicative of multiple acute cortical infarcts bilaterally, with the largest involving the posterior right MCA territory. CBF: Cerebral blood flow; CTH: CT of the head; CTP: CT perfusion; DWI: Diffusion-weighted MRI; MCA: Middle cerebral artery

Non-contrast CT of the head (CTH) confirmed wedge-shaped areas of hypoattenuation in the RMCA territory, compatible with acute infarcts involving the perirolandic cortex, lateral aspect of the right parietal lobe, dorsal aspect of the right superior temporal lobe, and adjacent posterior insular cortex (Figure 1A). CT angiography of head and neck demonstrated a distal right M3 occlusion with a matched perfusion deficit (Figure 1B). MRI performed one day later revealed multiple acute embolic infarcts in both cerebral hemispheres with the largest involving the posterior right MCA territory associated with gyral swelling and mild local mass effect (Figure 1C).

The patient exhibited purulent skin wounds on his limbs, signs of severe sepsis, and multiorgan dysfunction, including fever (maximum temperature: 102^o^F, 38.89^o^C), acute hypoxemic respiratory failure, renal failure, and hypotension requiring vasopressors. A physical examination was also notable for a new diastolic murmur, splinter hemorrhages, and other stigmata of infective endocarditis.

**Figure 2:**
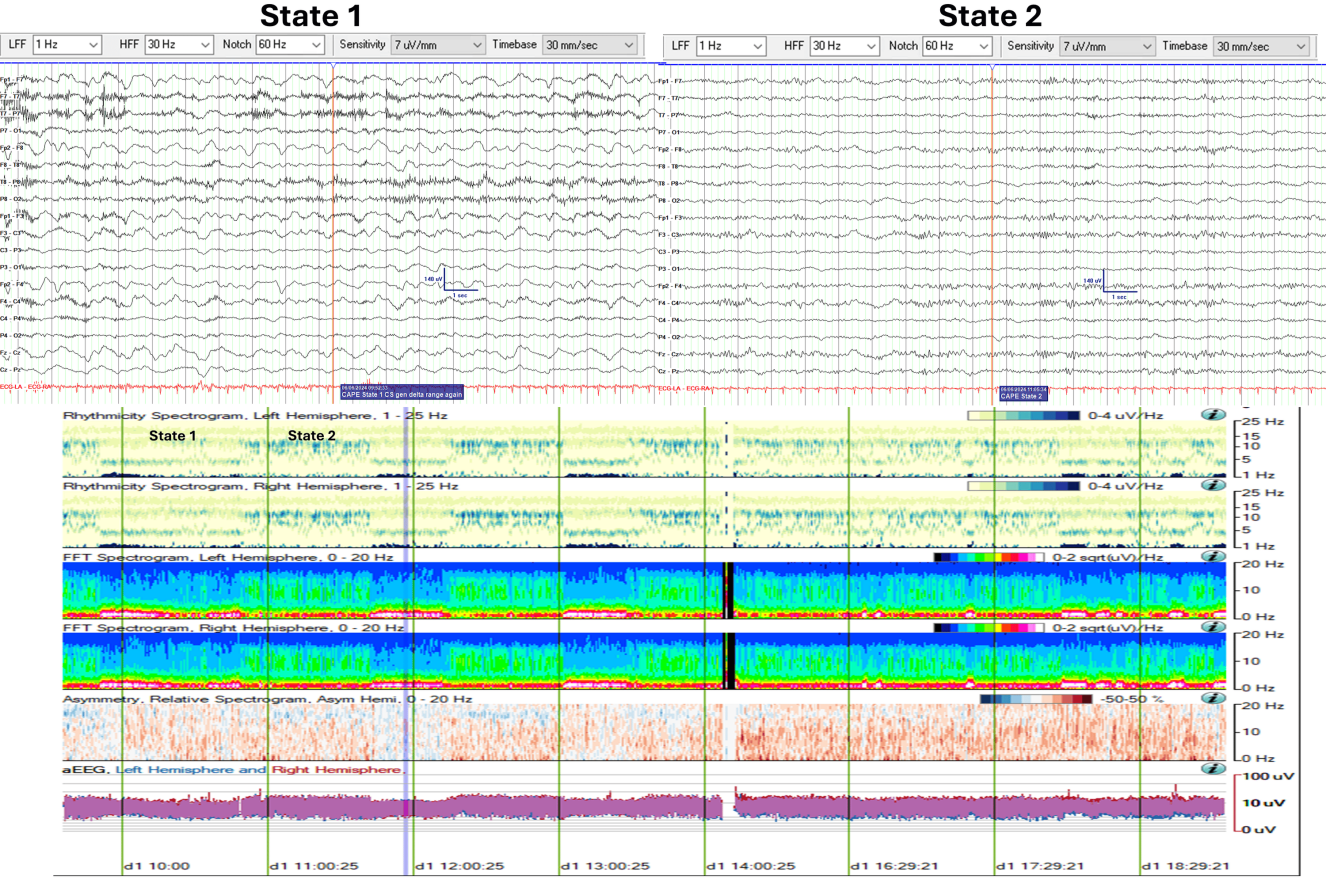
CAPE pattern in EEG and QEEG trends (Case 1) EEG and QEEG (eight-hour segment from 10:00 to 18:00) illustrating CAPE with two distinct states. State changes are evident in the rhythmicity and FFT spectrograms. State 1 shows increased spectral power density in the delta range frequencies (0-4 Hz), while State 2 demonstrates higher spectral power density in the theta-beta range frequencies (4-16 Hz). CAPE: Cyclic alternating pattern of encephalopathy; FFT: Fast Fourier transform; QEEG: Quantitative EEG

cEEG monitoring was initiated for neuromonitoring, revealing CAPE with two abnormal states. State 1 (Figure 2) was characterized by a continuous diffuse delta range (0.5-3.5 Hz, 30-60 uV) semi-rhythmical slow wave activity. State 2 (Figure 2) was characterized by continuous diffuse theta-alpha range (7-12 Hz, 20-45 uV) activity. Despite sedative pauses, the patient remained encephalopathic, as evidenced by opening his eyes to stimulation and localization to noxious stimuli, though he was unable to follow commands. Neuroimaging remained stable without the development of malignant cerebral edema.

Figure 2 displays an eight-hour QEEG (from 10:00 to 18:00) demonstrating the CAPE pattern. Visualized trends include rhythmicity spectrograms (left and right hemispheres), fast Fourier transform (FFT) spectrograms (left and right hemispheres), asymmetry spectrograms (left vs right), and amplitude-integrated EEG (aEEG, left vs right) graphs. Additionally, two 19-second EEG pages are taken from two time points corresponding to each abnormal state.

Initially, rhythmicity and FFT spectrograms revealed clearcut state changes occurring approximately every 40 minutes. State 1 demonstrated higher spectral power density associated with delta range frequencies (0-4 Hz). State 2 showed higher spectral power in the theta-beta range frequencies (4-16 Hz). The observed state changes were not noticeable over the asymmetry spectrograms or the aEEG trends.

Beginning at 13:30, State 2 persisted for an extended period of approximately four hours before the next state change was captured at 17:30.

Case 2

A 71-year-old female with advanced multiple sclerosis (MS), complicated by recurrent urinary tract infections (UTIs) and epilepsy, was admitted for evaluation of altered mental status, fever, and leukocytosis. Urine culture identified polymicrobial infection with *Serratia marcescens* and *Pseudomonas aeruginosa*. The patient was started empirically on broad-spectrum antibiotics, which were later escalated to include meropenem due to clinical deterioration.

**Figure 3:**
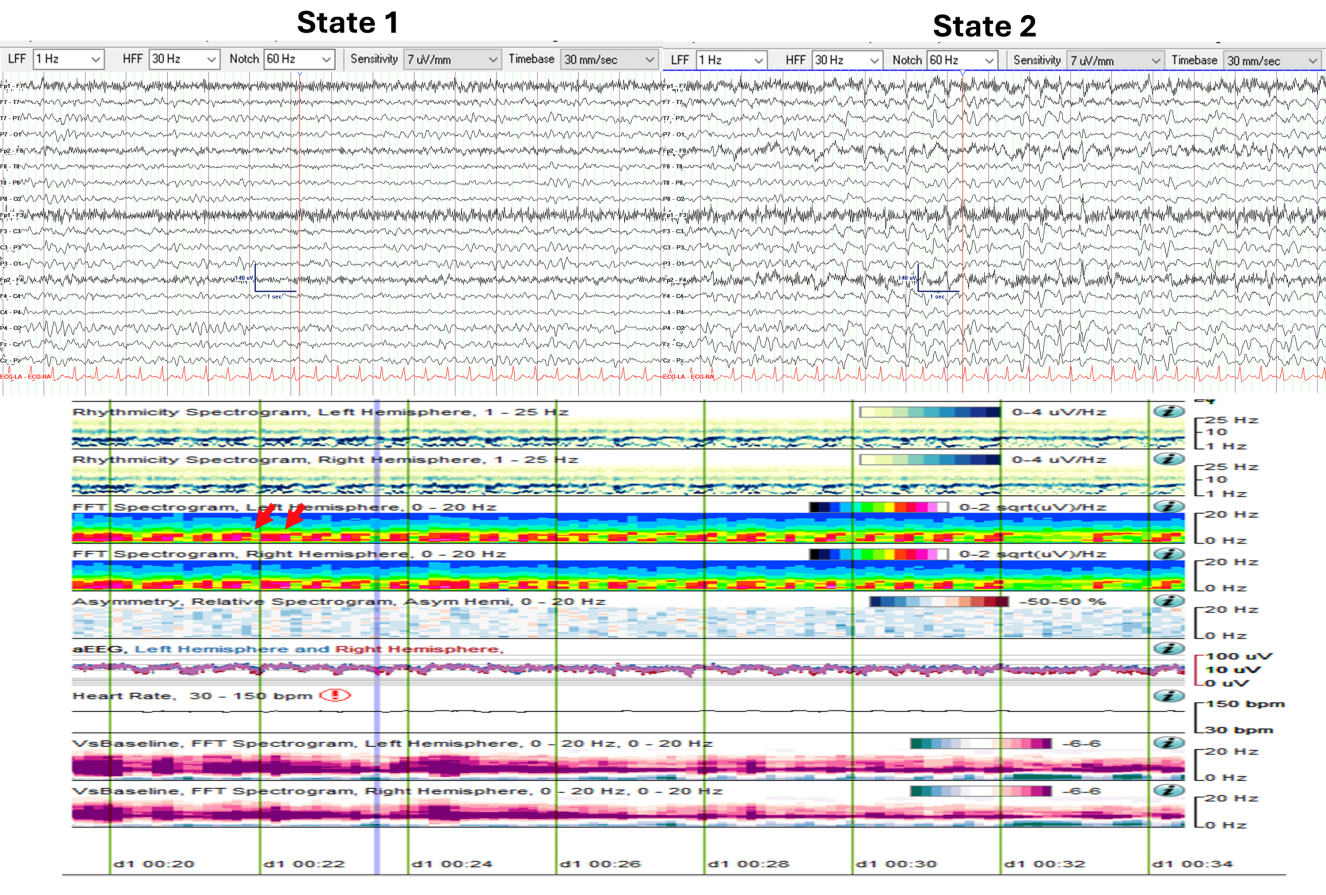
CAPE pattern in EEG and QEEG trends (Case 2) EEG and QEEG (15-minute segment from 00:20 to 00:35) demonstrating the CAPE pattern with two distinct states. State changes are highlighted by red arrows in the FFT spectrograms. In State 1, semi-rhythmic slowing is observed in the theta range (4-7 Hz). In State 2, there is increased spectral power in the delta range (1-4 Hz), corresponding to quasi-periodic triphasic waves. State changes are also evident in the aEEG trends, where amplitude is more than 50% higher in State 2 compared to State 1. aEEG: Amplitude-integrated EEG; CAPE: Cyclic alternating pattern of encephalopathy; FFT: Fast Fourier transform; QEEG: Quantitative EEG

The patient developed worsening encephalopathy and focal twitching of the left side of the face, arm, and leg, raising concerns for seizures. She was transferred to the ICU and intubated for anesthesia to manage status epilepticus. cEEG monitoring was initiated to evaluate for non-convulsive status epilepticus (NCSE) and titration of anti-seizure medications (ASM). cEEG demonstrated a CAPE pattern with two abnormal states: State 1 (Figure 3) was characterized by continuous generalized theta range (4-7 Hz, 20-40 uV) semi-rhythmical slowing, and State 2 (Figure 3) was characterized by continuous generalized delta-theta range (1-5 Hz, 20-50 uV) semi-rhythmical slowing. During State 2, intermixed generalized quasi-periodic discharges with triphasic morphology and anterior-to-posterior phase delay (2.5 Hz, 50-90 uV) were also observed.

Figure 3 presents a 15-minute QEEG segment (from 00:20 to 00:35) illustrating the CAPE pattern. Visualized trends include rhythmicity spectrograms (for both the left and right hemispheres), FFT spectrograms (for both hemispheres), asymmetry spectrograms (comparing left versus right), and aEEG (comparing left versus right) graphs. Additionally, two 19-second EEG snapshots are provided, each corresponding to different abnormal states.

Rhythmicity and FFT spectrograms clearly reveal state changes. In State 2, there was an increase in spectral power within the delta range frequencies (1-4 Hz), which correlates with the presence of quasi-periodic triphasic waves.

The state changes were also evident in the aEEG trends, where the amplitude in State 2, influenced by the presence of triphasic waves, was more than 50% higher than the background activity observed in State 1.

**Figure 4:**
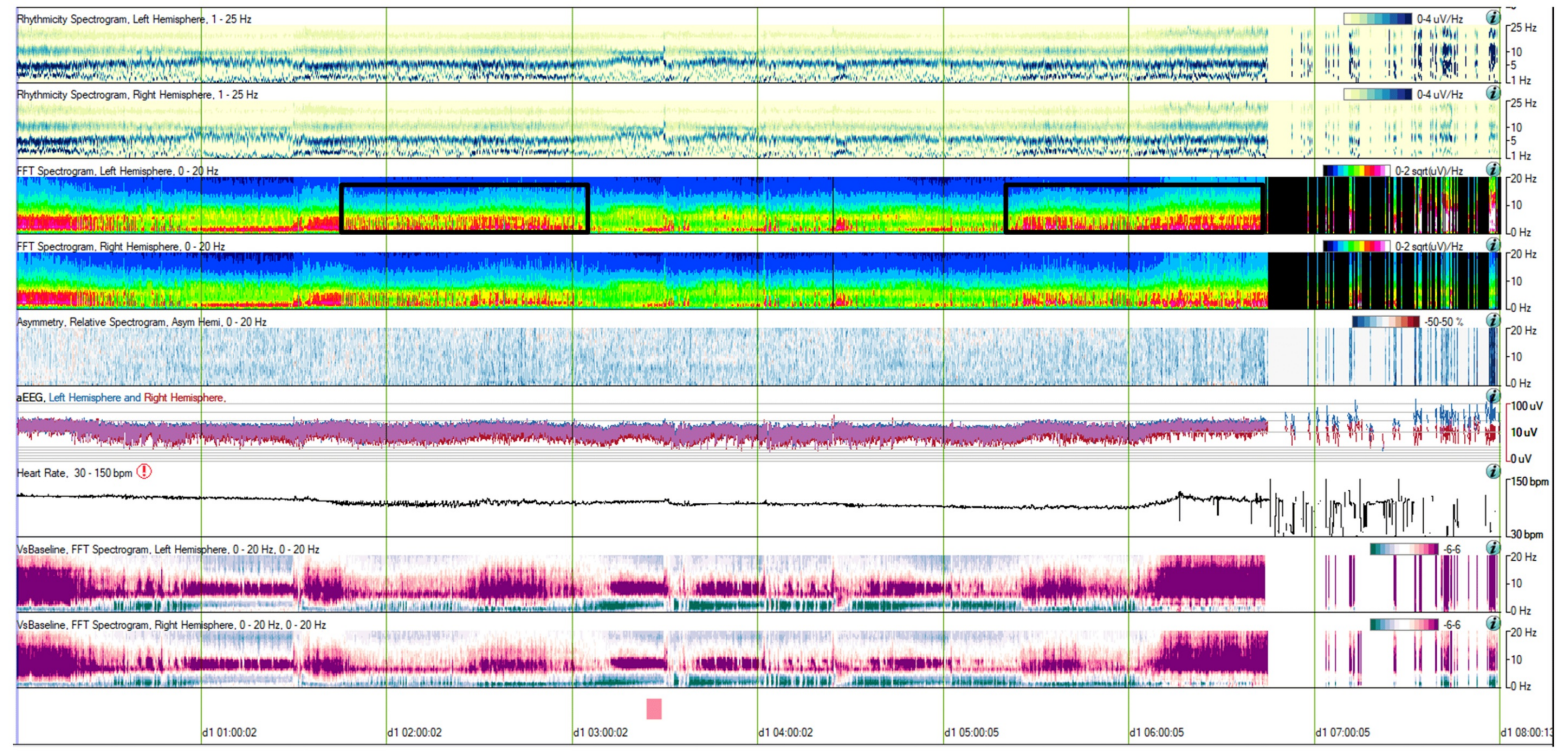
CAPE pattern in QEEG, extended temporal resolution (Case 2) QEEG (eight-hour segment from 00:00 to 08:00) illustrating prolonged stable states lasting up to 2.5 hours, followed by periods of rapid state fluctuation. Black boxes highlight the rapid fluctuation periods, where state changes occur every 30 to 60 seconds. CAPE: Cyclic alternating pattern of encephalopathy; QEEG: Quantitative EEG

QEEG analysis over a broader temporal resolution (8 hours, 00:00 to 08:00) revealed prolonged periods of stable states, lasting up to 2.5 hours. These were followed by periods of rapid state fluctuation, during which state changes occurred every 30 to 60 seconds and persisted for up to 1.5 hours (Figure 4).

Case 3

A 32-year-old male patient with OUD was found unresponsive at home. The day prior, he reported headaches, nausea, and vomiting. EMS administered Narcan in the field without improvement in mental status. He subsequently developed tonic-clonic movements concerning for seizures and was treated with lorazepam and subsequently intubated for airway protection and acute hypoxemic respiratory failure.

**Figure 5:**
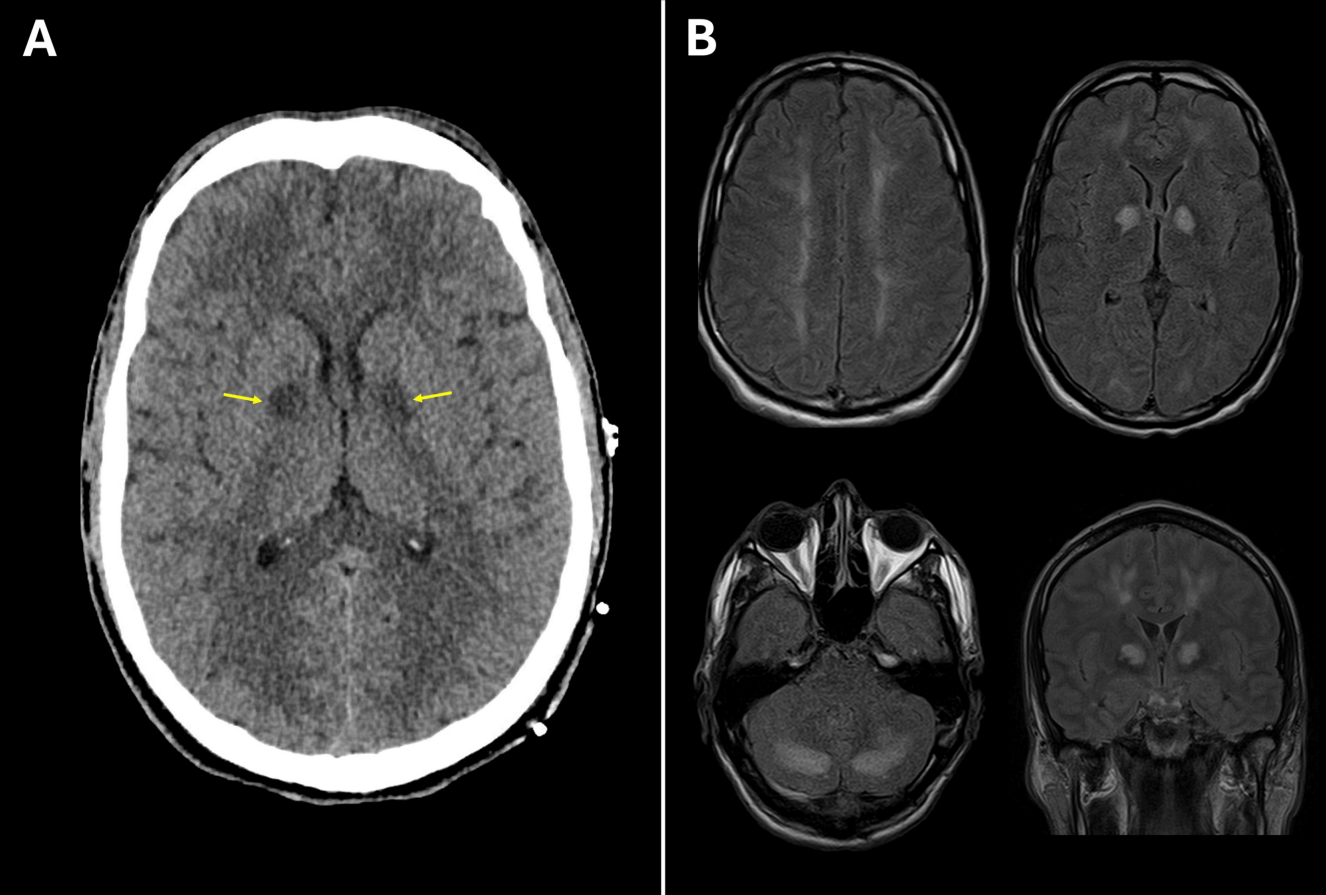
Neuroimaging (Case 3) (A) CTH demonstrating bilateral basal ganglia hypodensities. (B) MRI shows T2-FLAIR hyperintensities throughout the deep supratentorial white matter, posterior limbs of the internal capsules, subcortical white matter in the occipital lobes, and cerebellar white matter bilaterally, compatible with acute toxic leukoencephalopathy. CTH: CT of the head

Admission laboratory studies revealed a urine drug screen being positive for synthetic fentanyl and opioids. Admission CTH showed diffuse loss of grey-white matter differentiation and bilateral symmetric hypodensities within basal ganglia concerning for hypoxic ischemic injury (Figure 5A). Subsequent MRI of the brain was consistent with toxic leukoencephalopathy with symmetrical white matter T2-FLAIR hyperintensities and restricted diffusion, sparing the subcortical U-fibers but with significant mass effect and cortical vasogenic edema (Figure 5).

**Figure 6:**
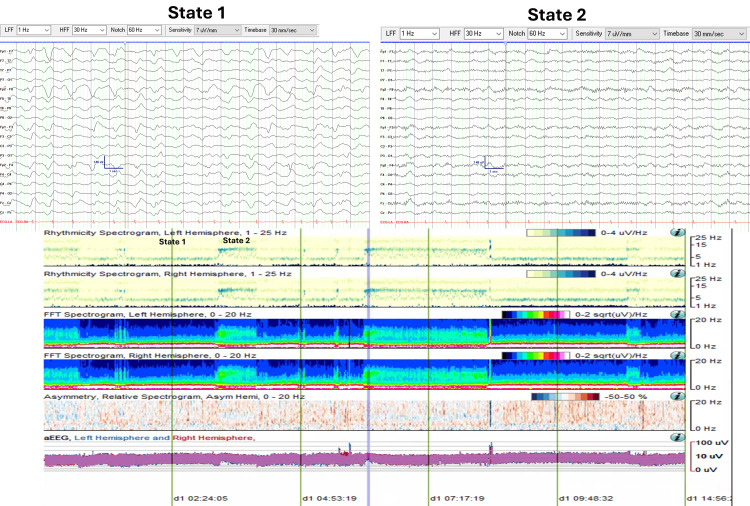
CAPE pattern in EEG and QEEG trends (Case 3) EEG and QEEG (15-hour segment from 00:00 to 15:00) demonstrating the CAPE pattern with two distinct states. State changes are visible in the rhythmicity and FFT spectrograms. State 1 exhibits higher spectral power density in the delta range (0-2 Hz). State 2 exhibits higher spectral power density in the alpha range (10-12 Hz). CAPE: Cyclic alternating pattern of encephalopathy; FFT: Fast Fourier transform; QEEG: Quantitative EEG

cEEG monitoring was initiated. Neurological examination revealed intact brain stem reflexes except for decreased pupillary reactivity bilaterally on quantitative pupillometry. The patient was treated empirically with levetiracetam, but no seizures were captured on cEEG. Instead, a CAPE pattern was observed with two abnormal states. State 1 (Figure 6) was characterized by continuous diffuse delta-range (0.5-1.5 Hz, 40-140 µV) slow-wave activity. State 2 (Figure 6) was characterized by continuous diffuse delta-range (0.5-1.5 Hz, 30-60 µV) slow-wave activity with superimposed diffuse alpha-range activity (11-12 Hz, 10-40 µV).

Figure 6 displays a 15-hour QEEG segment (00:00 to 15:00) illustrating the CAPE pattern. The rhythmicity and FFT spectrograms demonstrate state changes. In State 1, there was increased spectral power density in the delta range (0-2 Hz), while in State 2, the higher spectral power shifted to the alpha range (10-12 Hz). The state changes were not discernible in the asymmetry spectrograms or the aEEG trends. However, the two states are not discernible in the asymmetry or aEEG trends.

Case 4

A 72-year-old female with severe pulmonary and tricuspid valve regurgitation and a pulmonary artery aneurysm presented to the hospital in cardiogenic shock. Following resuscitation, she underwent redo sternotomy for pulmonary valve replacement, tricuspid valvuloplasty, and pulmonary arterioplasty. Postoperatively, the patient developed renal failure requiring continuous renal replacement therapy (CRRT). She subsequently exhibited abnormal, episodic, and irregular myoclonic movements involving the face, limbs, and torso. Given her post-surgical sedation, cEEG monitoring was initiated to evaluate for acute symptomatic seizures.

**Figure 7:**
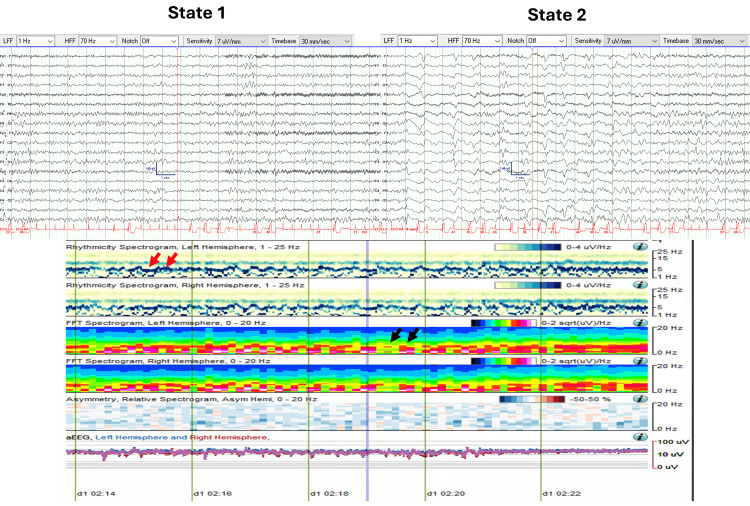
CAPE pattern in EEG and QEEG trends (Case 4) EEG and QEEG (10-minute segment from 02:14 to 02:24) illustrating the CAPE pattern with two distinct states. State changes are visible in the rhythmicity and FFT spectrograms. Red arrows indicate an upward deflection of theta-alpha activity in State 1. Black arrows highlight increased spectral power in the delta range (1-4 Hz) during State 2, correlating with triphasic waves. CAPE: Cyclic alternating pattern of encephalopathy; FFT: Fast Fourier transform; QEEG: Quantitative EEG

cEEG revealed a CAPE pattern with two abnormal states. State 1 (Figure 7) was characterized by continuous generalized theta-alpha (6-7 Hz, 20-40 µV) semi-rhythmic slowing. State 2 (Figure 7) was characterized by continuous generalized delta-theta range (0.5-5 Hz, 40-75 µV) slowing, intermixed with generalized periodic discharges with triphasic morphology.

Figure 7 presents a 10-minute QEEG segment (02:14 to 02:24) illustrating the CAPE pattern. The rhythmicity and FFT spectrograms demonstrate state changes occurring every 10 to 120 seconds. During State 1, there was increased power associated with theta-alpha range frequencies. This is visible as an upward deflection of the theta-alpha activity on the rhythmicity spectrograms. In State 2, there was increased spectral power in the delta range (1-4 Hz), which also correlated with the presence of quasi-periodic triphasic waves.

**Figure 8:**
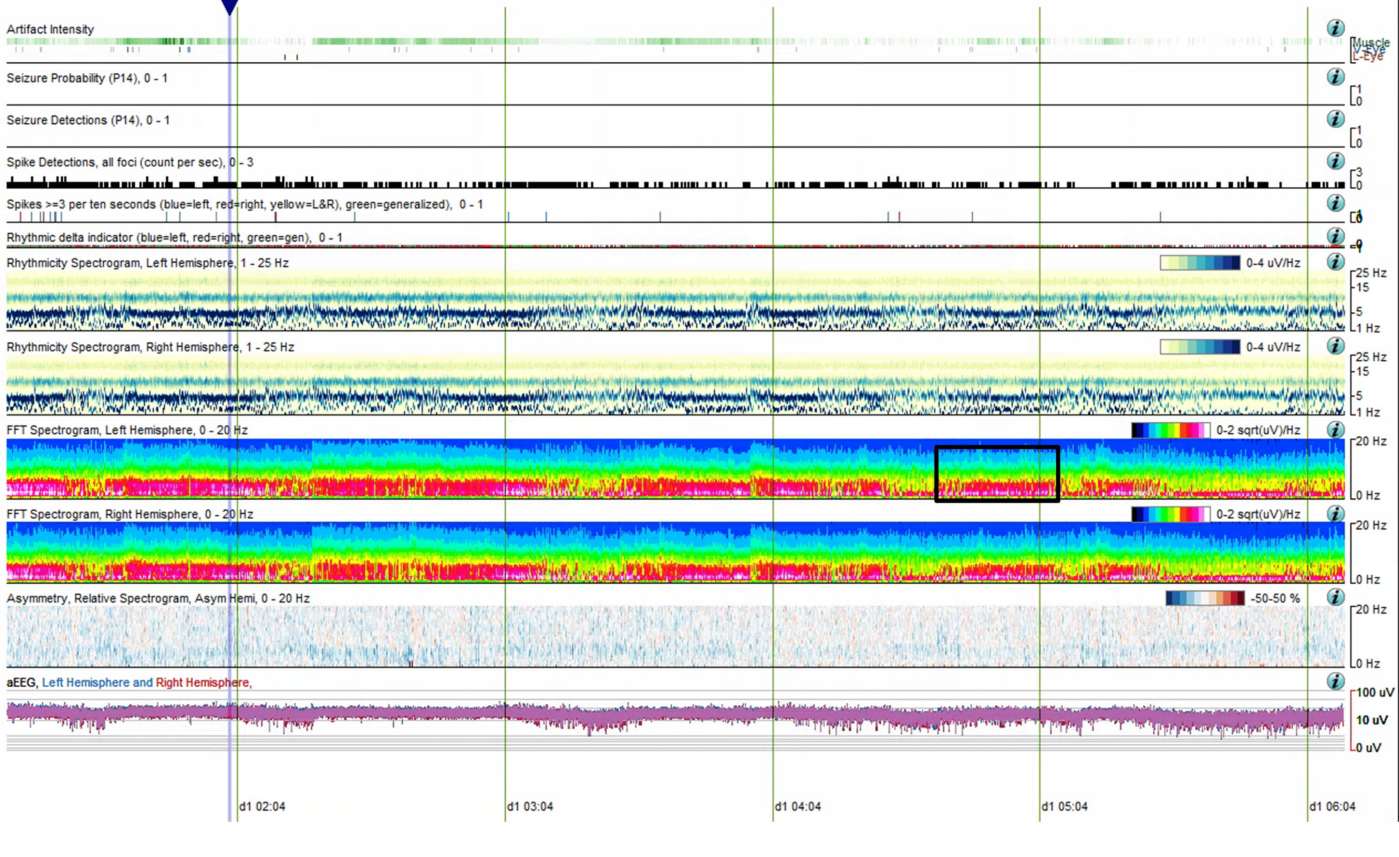
CAPE pattern in QEEG, extended temporal resolution (Case 4) QEEG (six-hour segment from 00:00 to 06:00) demonstrating extended periods of alternating states. The black box highlights periods of rapid state fluctuations, with changes occurring every 10 to 120 seconds. CAPE: Cyclic alternating pattern of encephalopathy; QEEG: Quantitative EEG

Similar to Case 2, a longer QEEG segment (six hours, 00:00 to 06:00) demonstrated extended periods of stable states lasting up to 1.5 hours. Periods of rapid state fluctuation, with changes occurring every 10 to 120 seconds, were also observed (Figure 8).

## Discussion

We present four cases of encephalopathic patients with varying acute brain insults monitored with cEEG. These patients exhibited the CAPE pattern. The QEEG features of CAPE are highlighted for each case. Changes in rhythmicity, asymmetry, and the color density spectral array proved effective in identifying CAPE. We also demonstrated how adjusting the temporal resolution of QEEG visualizations improves the visibility of CAPE patterns across both longer and shorter timeframes.

QEEG is a valuable tool for identifying CAPE. By compressing time, QEEG allows for rapid analysis of large datasets. Typical trends in QEEG are based on Fourier domain analysis, which breaks down EEG signals into their frequency components and visualizes them by their power or amplitude. In the presented FFT trends, cooler colors represent lower amplitude/power, while warmer colors indicate higher amplitude/power. The rhythmicity trend, derived from similar frequency decomposition, highlights periodicity and sometimes provides superior visualization of state changes. Asymmetry trends compare the power and frequency distribution between the left and right hemispheres, aiding in the detection of imbalances or irregularities.

QEEG has been applied in epilepsy management and neurocritical care settings. Noteworthy applications include the automated detection of epileptiform activity and seizures through algorithms based on FFT trends and spike-detection methods, which enhance sensitivity in identifying NCSE [13]. Additionally, QEEG has been employed for the early detection of ischemic changes [14,15]. In another context, it has been utilized to evaluate quantitative changes associated with Brivaracetam therapy in patients with drug-resistant epilepsy, highlighting its capability to measure slow, time-varying alterations [16]. Despite these diverse applications, limitations exist. The accuracy of seizure detection and trend interpretation relies heavily on the expertise of the interpreter [13], as incorrect or inconsistent readings can lead to missed detections or false positives.

The clinical significance of CAPE in encephalopathic patients remains uncertain. We hypothesize that CAPE may represent a finding associated with brain recovery. Previous studies have shown that EEG reactivity to external stimuli is a favorable prognostic marker in critically ill patients [17]. CAPE may be a form of reactivity or arousal. It is observed to occur in various pathologies as we demonstrate here. The presence does not seem to reflect adjustment in sedation. However, we acknowledge that our observations are based on a small sample of cases, which limits the statistical power and generalizability of our conclusions. Larger studies are needed to validate these observations.

Additionally, current QEEG guidelines for clinical practice underscore the lack of predictive metrics, emphasizing that its explanatory value often surpasses its predictive capabilities [18]. This highlights the need for further validation and standardization, although emerging trends in multivariable and machine learning applications show promise [19,20].

As mentioned earlier, further research is needed to better define the role of CAPE in critically ill patients. Investigating the presence or absence of CAPE across diverse brain insults and patient outcomes could help determine its potential as a prognostic biomarker. CAPE’s association with dynamic state transitions suggests it may provide valuable insights into underlying pathophysiological mechanisms and prognosis in encephalopathy.

## Conclusions

CAPE is a frequent finding on cEEG monitoring, reflecting state changes in encephalopathic patients. This case series highlights the utility of QEEG in identifying the CAPE pattern through the visualization of FFT trends, rhythmicity spectrograms, aEEG, and asymmetry spectrograms. Unlike raw cEEG, which provides time domain data, QEEG can be used to readily identify and quantify the duration and frequency of multiple state transitions through time-compressed trends. By visualizing EEG data across varying temporal resolutions, QEEG enhances the detection and characterization of CAPE.

The detailed visualization of CAPE patterns provides a unique perspective on the temporal and spectral dynamics of brain activity during critical illness. By elucidating these dynamic state transitions, QEEG contributes to a more comprehensive understanding of encephalopathic states. Further research should explore the prognostic value of CAPE and its potential implications for patient management and outcomes in the critical care setting.
